# Mitigation of Single-Event Effects in SiGe-HBT Current-Mode Logic Circuits

**DOI:** 10.3390/s20092581

**Published:** 2020-05-01

**Authors:** Md Arifur R. Sarker, Seungwoo Jung, Adrian Ildefonso, Ani Khachatrian, Stephen P. Buchner, Dale McMorrow, Pauline Paki, John D. Cressler, Ickhyun Song

**Affiliations:** 1 School of Electrical and Computer Engineering, Oklahoma State University, Stillwater, OK 74078, USA; msarker@okstate.edu; 2 Broadcom Inc., San Jose, CA 95131, USA; jung@gatech.edu; 3 School of Electrical and Computer Engineering, Georgia Institute of Technology, GA 30318, USA; iadrian@gatech.edu (A.I.); cressler@ece.gatech.edu (J.D.C.); 4 US Naval Research Laboratory, Washington, DC 20375, USA; ani.khachatrian@nrl.navy.mil (A.K.); stephen.buchner@nrl.navy.mil (S.P.B.); dale.mcmorrow@nrl.navy.mil (D.M.); 5 Defense Threat Reduction Agency, Fort Belvoir, VA 22060, USA; pauline.f.paki.civ@mail.mil

**Keywords:** current-mode logic (CML), heterojunction bipolar transistor (HBT), negative feedback, radiation hardening by design (RHBD), single-event effects (SEE), single-event transients (SET), silicon germanium (SiGe)

## Abstract

It has been known that negative feedback loops (internal and external) in a SiGe heterojunction bipolar transistors (HBT) DC current mirrors improve single-event transient (SET) response; both the peak transient current and the settling time significantly decrease. In the present work, we demonstrate how radiation hardening by design (RHBD) techniques utilized in DC bias blocks only (current mirrors) can also improve the SET response in AC signal paths of switching circuits (e.g., current-mode logic, CML) without any additional hardening in those AC signal paths. Four CML circuits both with and without RHBD current mirrors were fabricated in 130 nm SiGe HBT technology. Two existing RHBD techniques were employed separately in the current mirrors of the CML circuits: (1) applying internal negative feedback and (2) adding a large capacitor in a sensitive node. In addition, these methods are also combined to analyze the overall SET performance. The single-event transients of the fabricated circuits were captured under the two-photon-absorption laser-induced single-event environment. The measurement data clearly show significant improvements in SET response in the AC signal paths of the CML circuits by using the two radiation hardening techniques applied only in DC current mirrors. The peak output transient current is notably reduced, and the settling time upon a laser strike is shortened significantly.

## 1. Introduction

Silicon–germanium (SiGe) heterojunction bipolar transistors (HBTs) have received extensive attention for implementation in extreme-environment applications due to their excellent total-ionizing-dose (TID) tolerance, high-speed operation (i.e., high unity gain frequency, *f*_T_), superb cryogenic performance, and ease of integration in a complementary metal oxide semiconductor (CMOS) platform [1,2,3,4]. However, studies have reported that SiGe HBTs are susceptible to single-event phenomena, especially single-event upset (SEU) and single-event transient (SET) [5,6,7], and thus, it is highly critical that the sensitivity of SiGe HBT circuits to single-event effect (SEE) be improved, either via changing the underlying device profile, i.e., radiation hardening by process (RHBP) or circuit design, i.e., radiation hardening by design (RHBD), or both. Developing radiation-hardened platforms require significant effort to optimize and are very expensive. As a result, RHBD techniques are preferred for hardening solution [8]. For example, it has been demonstrated that the current-mode logic (CML) circuit is used as one of the core building blocks of high-speed integrated-circuit (IC) applications such as analog-to-digital converters (ADCs), digital-to-analog converters (DACs), voltage-controlled oscillators (VCOs), and frequency synthesizers [9,10]. Typical CML circuits consist of two main parts, the AC differential signal path and the DC current source, in which a current-mirror structure is commonly implemented [11,12]. The bias circuits have proven to be very sensitive to single-event effects (SEEs) in many different analog and mixed-signal applications, since they affect the overall operation conditions of circuits [13,14,15]. As a RHBD solution to mitigate SET, a negative feedback in DC SiGe HBT current mirrors was proposed and a significant reduction in the peak transient and settling time was also achieved [12].

The SEE on CML in metal-oxide-semiconductor (MOS) technologies has been studied in detail over the years. Regarding SiGe HBT-based CML, an extensive analysis regarding the physics of SEU is presented in [16,17,18]. As an RHBD approach, a technique that actively cancels charge in a sensitive node was proposed and demonstrated by Armstrong et al. [19]. Different RHBD techniques have been proposed to mitigate SEEs in CML circuits such as device level mitigation [20,21,22], demonstrating the use of p-n-p SiGe HBTs, transistor level layout modification, and shared dummy collector for SEU mitigation. Krithivasan et al. proposed the dual-interleaving and gated-feedback techniques combined, also incorporating the triple modular redundancy (TMR) technique as circuit level SEU mitigation [23] at the cost of a very high-power and area penalty. Recently, it has been demonstrated that SiGe HBTs in inverse-mode operation have improved SET response in analog and high-frequency applications [24,25,26]. However, due to the large parasitics associated with the inverse-mode operation of SiGe HBTs, significant high-frequency performance degradation occurs. 

In the present work, we investigate how RHBD techniques that are optimally utilized in ubiquitous DC-bias blocks can also improve the SET response in the AC path of CML circuits, i.e., no additional hardening is needed in the AC path for SET mitigation. In comparison with the findings in [4], where the mitigation of SETs was observed only at the output of the DC-bias block itself, here we propose that the radiation-hardening of the *DC-bias circuitry only* also results in better SET generated *within AC CML circuits* in terms of transient peak and duration. This is more attractive in terms of AC operation, since the direct application of hardening techniques to AC (or high-frequency) circuits is likely to lead to a tradeoff of performance degradation such as gain reduction, a noise increase, etc. Hence, the outcome of this investigation can be applicable to a broad range of circuits and systems without significant changes in high-frequency performance.

The rest of the paper is organized as follows. Section 2 describes the operation of CML circuits and the applied RHBD techniques in the DC current-mirror part of a CML block. Section 3 explains the experimental setup, and Section 4 presents the measurement and the simulation results. Finally, Section 5 remarks on the conclusions.

## 2. Current-Mode Logic Circuits

**Figure 1:**
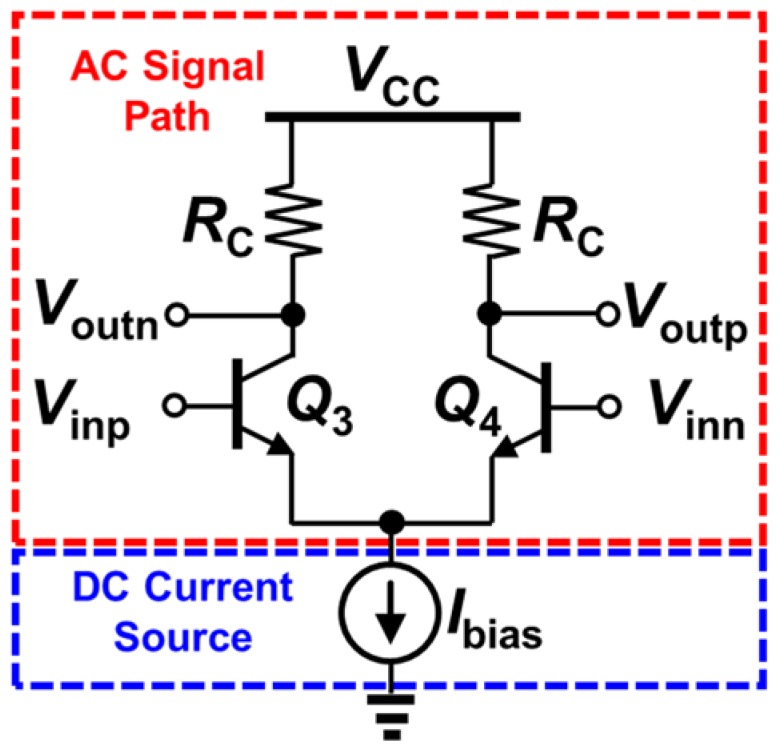
Current-mode logic (CML) circuit consists of an AC signal path (differential input pair) and a DC current source.

Current-mode logic (CML) circuits are a fundamental building block in numerous high-speed IC applications. They are composed of two main pieces: an AC differential pair and a DC bias current source (e.g., current mirror), as shown in Figure 1. Unlike standard low-power consumption logic circuits, CML circuits are always “on”—That is, either side of the differential transistor pair always remains on, thus constantly pulling current from the source. Thus, CML circuits can operate at very high frequencies, since the primary component of delay, caused by the turn-on and turn-off time or the requisite devices, is avoided in the circuit operation (the devices are always turned on). The drawback, of course, is that the power consumption of CML circuits is greater than that of the standard logic circuits. The load resistor R_C_ is optimized for matching, voltage swing, or delay.

**Figure 2:**
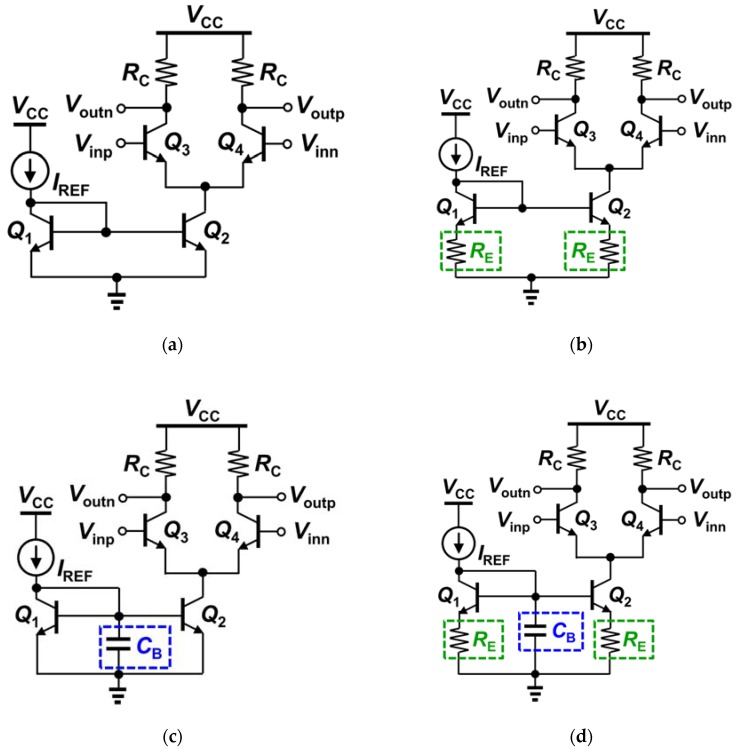
Four CML circuits are fabricated and investigated. Radiation hardening by design techniques using internal negative feedback created by the emitter degeneration resistor R_E_ (100 Ω) and the capacitor C_B_ (12 pF) are applied to the DC current mirrors in the three of four CML circuits (**b**–**d**), with (**a**) served as a control.

The AC signal path in CML circuits is minimally affected by the tail source at a specific DC bias condition at low frequencies where the impact of impedance variations is negligible (typically, < 10 GHz or higher in advanced technology nodes). However, as the operating frequency increases, the AC differential pairs and the DC current mirrors begin to interact with each other through parasitic components (both capacitive and inductive). It has been shown that the SET response can be significantly improved in DC current mirrors by applying SET reduction techniques such as internal and external negative feedback [4]. Since these SET reduction techniques in DC current mirrors are devised to regulate (or suppress) the abrupt AC transient due to single-event phenomena, it is expected that these design techniques within the current mirrors can also improve the SET response through the AC signal path in CML circuits by preventing or suppressing the crosstalk at higher frequencies. In order to validate these claims, four SiGe CML circuits were investigated, as shown in Figure 2.

As SET hardening, two hardening techniques utilized in the fabricated CML circuits include an internal negative feedback loop and the addition of a large capacitor. Negative feedback has been proven to lessen the impact of SETs in electronic systems, thereby helping their rapid recovery because it decreases the sensitivity of a system to abrupt changes. Capacitors can be used in forming a low-pass filter, which mitigate undesirable high-frequency transients. In fact, it is a common practice to add large capacitors on supply rails in order to filter out abrupt transients so that remaining electronics do not malfunction. Another way of looking at this from the time-domain perspective is that since the voltage across a capacitor cannot change instantaneously, it mitigates unwanted rapid transients; thus, low-pass filters are also referred to as smoothing filters. 

The internal negative feedback is created by adding the degeneration resistor R_E_ (100 Ω) at the emitters of the current mirror (Figure 2b). The capacitor (12 pF) is connected between the base of the reference device (Q_1_) of the current mirror and ground (Figure 2c). Since the voltage across a capacitor cannot change instantaneously, as shown in Equation (1), the rapid transient of the base voltage of the reference device due to SEE can be effectively reduced. Here, *i_Cap_*(*t*) is the current through the capacitor, *v_Cap_*(*t*) is the voltage across the capacitor, and C is the capacitance.

(1) $$i_{Cap}\left(t\right)=C\frac{dv_{Cap}\left(t\right)}{dt}$$

## 3. Measurement Setup

**Figure 3:**
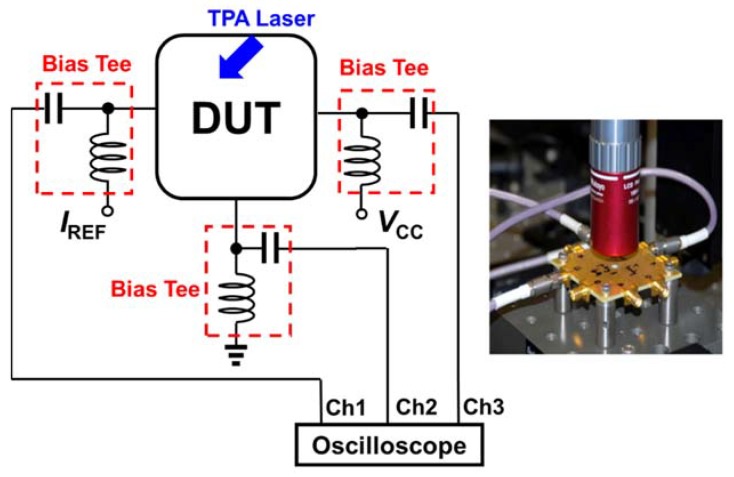
Diagram of the experimental measurement configuration and a photograph of the actual setup.

The single-event transient (SET) measurements were conducted at the U.S. Naval Research Laboratory using a two-photon-absorption (TPA) pulsed-laser single-event effect through the wafer technique [3,4]. The configuration diagram is depicted in Figure 3, together with monitoring terminals. For the present investigation, four CML circuits with hardened and unhardened DC bias blocks (current mirrors) were fabricated using a commercial 130 nm SiGe HBT technology. The devices under test were packaged using a high-speed custom-designed printed circuit board, high-frequency connectors, low-loss cables, DC power supplies, and 67 GHz-capable bias tees. Resulting SETs were captured through a Tektronix high-speed DPO71254 12.5 GHz real-time oscilloscope. In addition, Figure 3 shows a photograph of the actual measurement setup.

## 4. Results and Discussion

The current mirror in the reference CML circuit, as shown in Figure 2a, does not utilize any radiation-hardening techniques. The internal negative feedback created by the emitter degeneration resistor R_E_ is employed in the current mirror of the second CML circuit (Figure 2b). The mirror in the third CML circuit (Figure 2c) utilizes the capacitor between the base of the reference device Q_1_ and ground. In the fourth CML circuit, both the internal feedback and the capacitor are used in order to improve the SET response of the circuit. In all cases, the supply voltage V_CC_ was set to 2.2 V, the reference current I_ref_ was set to 200 µA, and R_C_ was chosen to be 50 Ω. 

**Figure 4:**
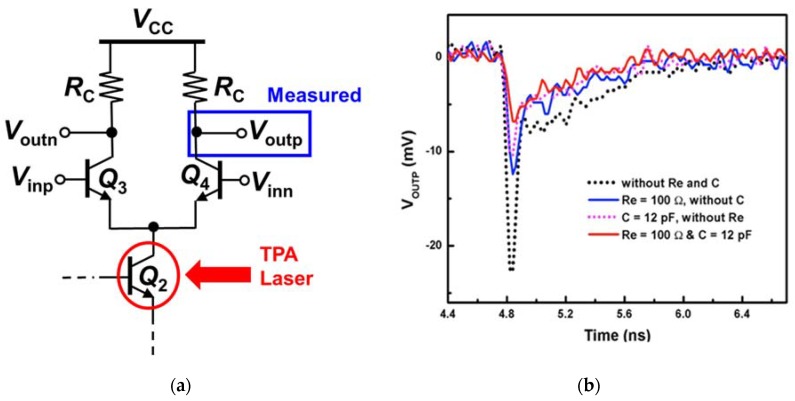
(**a**) The output transistor Q_2_ of the DC current mirrors of all four CML circuits in Figure 2 was struck by the two-photon-absorption (TPA) laser; (**b**) Transient responses at the positive output terminal (Q_4_ collector) of the differential pair was measured.

First, the output transistor Q_2_ of the DC current mirrors of all four CML circuits in Figure 2 was struck by the TPA laser (Figure 4a), and the transient response at the differential output terminals was measured. The internal negative feedback created by the emitter degeneration resistor R_E_ (100 Ω) in Figure 2b, as reported in Jung et al. [12], improves the SET response of the CML circuit. As a result, the peak transient of the output signal of the CML circuit (solid blue line in Figure 4b) is reduced by 50% compared to the control circuit (dotted black line). The capacitor (12 pF) connected between the base of the reference device of the current mirror (Q_1_) and ground in Figure 2c reduces the peak transient of the output signal of the CML circuit when the output transistor Q_2_ of the DC current mirror is struck by the TPA laser; the peak transient is decreased by about 50% (dotted magenta line in Figure 4) compared to that of the CML control circuit (dotted black line). This substantial reduction in SET occurs due to the fundamental nature of capacitors; that is, the voltage across a capacitor cannot change instantaneously. When device Q_2_ is struck by the TPA laser, the voltage at the base node of Q_1_ tends to undergo a strong current transient. However, the capacitor prevents this fast transient, endeavoring to maintain the original potential across itself. The best SET improvement is achieved by the CML circuit with the current mirror employing both the internal negative feedback and the capacitor, as shown by the solid red line of Figure 4b. The internal negative feedback together with the capacitor suppresses the peak transient of the output signal by 70% (red solid line in Figure 4).

**Figure 5:**
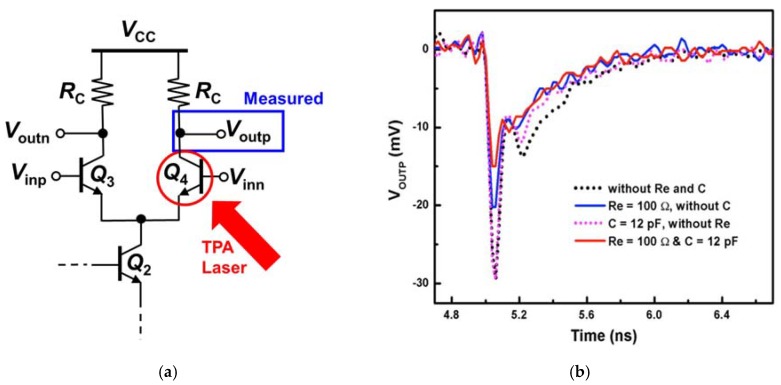
(**a**) Transistor Q_4_ of the differential input pair of all four CML circuits in Figure 2 was struck by the TPA laser; (**b**) Transient responses at the positive differential output terminal (Q_4_ collector) was measured.

In order to investigate the effects of these radiation hardening by design techniques implemented in the DC current mirrors on the AC signal path of the CML circuits, the differential input device of the CML circuits (Q_4_) was also struck by the TPA laser (Figure 5a). Figure 5b shows the measured transient responses of the output signal of the CML circuits when the input device Q_4_ is struck by the TPA laser. It is clearly shown that the internal negative feedback created by the emitter resistor R_E_ reduces the peak output transient by 35% (solid blue line in Figure 5b) compared to the CML control circuit (dotted black line). The transient current induced by the laser strike in Q_4_ flows through Q_2_ and the degeneration resistor R_E_. This transient current increases the emitter voltage of Q_2_ (*v_E2_*), resulting in a decrease in the base-emitter voltage of Q_2_ (*v_BE2_*). Thus, the collector current of Q_2_ decrease, as does the collector current of Q_4_ based on the collector current Equation (2); *I_S_* is the reverse saturation current, *V_T_* is the thermal voltage (which is about 26 mV at room temperature), and *V_A_* is early voltage. This negative feedback effect reduces the peak transient as well as the transient time.

(2) $$i_{C}=I_{S}e^{\frac{v_{BE}}{V_{T}}}\left(1+\frac{v_{CE}}{V_{A}}\right)$$

Interestingly, however, adding the capacitor alone in the current mirrors does not improve the SET response of the AC signal paths in the CML circuits (dotted magenta line in Figure 5b) compared to the CML control circuit (dotted black line). This is because the transient current induced by the laser current in Q_4_ flows through Q_2_ directly into the ground. In other words, the capacitor can only mitigate the rapid transient in the base node of Q_2_. However, the induced current by the laser strike does not cause a significant transient (or fluctuation) at the base when it flows through the device Q_2_ (even if it increases the base current slightly). The capacitor does not form any negative feedback loop, either.

As in the four cases in which the output device Q_2_ of the DC current mirrors was struck by the laser, the best SET improvement in the AC signal paths was achieved also in the CML circuit with the current mirror that employed both the internal negative feedback and the capacitor, as shown in the solid red line of Figure 5b. The internal negative feedback, together with the capacitor, most effectively suppresses the SET in the AC path (by about 50%). The same negative feedback mechanism applies in the CML circuit with the degeneration resistor R_E_ and the capacitor as in the CML circuit with only R_E_. The base voltage of Q_2_ (*v_B_*_2_) tends to increase as the emitter voltage (*v_E_*_2_) rapidly increases due to the base-emitter parasitic capacitance. This is, again, because the voltage across a capacitor cannot change instantaneously (the fundamental nature of a capacitor). However, this transient at the base of Q_2_ is suppressed by the much bigger capacitor connected between the base and the ground (C_B_ in Figure 2d). This is possible because one end of the capacitor C_B_ is connected to the ground (i.e., fixed DC voltage node); in other words, if the capacitor were floating, the further SET improvement shown in Figure 5 (solid red line) would be less effective.

**Figure 6:**
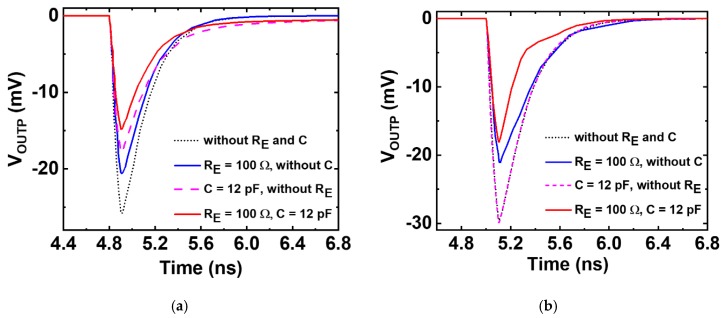
(**a**) Transient responses at the output terminal (Q_4_ collector) of the differential pair when an excess current was injected in the output transistor Q_2_ of the DC current mirrors of all four CML circuits in Figure 2; (**b**) Transient responses at the output terminal (Q_4_ collector) when an excess current was injected in transistor Q_4_ of the differential input pair of all four CML circuits in Figure 2.

The experimental results were also verified by the using double-exponential current-injection method [27,28]. The double-exponential current source was modeled following the SET response of 130 nm SiGe HBTs described in [29,30]. The simulated waveforms of the AC signal path due to a strike in the DC current-mirror transistor (Q_2_) and the differential-pair transistor (Q_4_) are shown in Figure 6. The simulation shows the similar trends observed from the experiment. When the excess current was injected at the Q_2_ transistor, it showed a reduction of 55% in transient peak when applying both R_E_ and C. The other two RHBD techniques also reduced transient peaks of 25% (R_E_ = 100 Ω, without C) and 35% (C = 12 pF, without R_E_), respectively. Similarly, when the current was injected in the Q_4_ transistor, the simulation results agree with the experimental data. The RHBD techniques of (1) using both R_E_ and C and (2) using R_E_ only without C exhibited 40% and 30% reductions in transient peaks, respectively. However, employing C only did not reduce transient peaks effectively, which was consistent with the experimental results. It should be noted that there are differences in the peaks and duration between the experimental results and simulation data due to limited accuracy in a double-exponential model and the current-injection method.

The experiment and simulation results clearly demonstrate that employing RHBD such as applying negative feedback and adding a capacitor in the DC bias blocks can significantly improve SET response even in the AC signal paths. This is a very encouraging result for circuit designers because it is extremely challenging to apply RHBD techniques to AC signal paths in high-speed applications without degrading their overall AC performance, especially as their operating frequencies increase. On the contrary, applying these hardening techniques to a DC bias block has a minimal impact on high-frequency AC performance. Due to the use of relatively large capacitance (12 pF), an obvious penalty induced by the use of these RHBD techniques is increased chip area. The overall performance metrics of the CML with and without the RHBD techniques are summarized in Table 1.

## 5. Conclusions

This work demonstrates that the SET response in AC signal paths in SiGe HBT CML circuits can be significantly improved by applying RHBD techniques in DC bias blocks such as current mirrors. This is an important finding because it shows that designers can avoid AC performance degradation while simultaneously radiation hardening their circuits. The internal negative feedback created by the 100 Ω emitter degeneration resistor in the current mirrors reduces the peak output transient of the CML circuit by 50% when the output device Q_2_ of the current mirror is struck and by 35% when one of the differential input device Q_4_ is struck by the TPA laser. The 12pF capacitor connected between the base of the reference device Q_1_ of the current mirror and ground decreases the peak output transient of the CML circuit by 50% when the output device Q_2_ of the current mirror is struck by the TPA laser. However, the capacitor alone does not improve the SET response of the output signal of the CML circuits when Q_4_ is struck by the TPA laser. The best result in terms of SET response improvement in the CML circuits is achieved by the combination of the negative feedback and the capacitor together in the cases where the DC device Q_2_ is struck and when the AC device Q_4_ is struck by the TPA laser; the peak output transient of the CML circuits is reduced by 70% when Q_2_ is struck and by 50% when Q_4_ is struck by the TPA laser, in circuits with both internal negative feedback and the capacitor utilized in the DC current mirrors.

## Figures and Tables

**Table 1:** Comparison of CML variants.

	Normalized 3-dB Bandwidth	Normalized Chip Area *	SET Generation	Normalized Transient Peak
Control No R_E_ and C	1 ^x^	1	CM ^+^ (Q_2_)	1
			Diff-pair (Q_4_)	1
R_E_ = 100 Ω, C = 0 pF	1.02	2.4	CM (Q_2_)	0.50
			Diff-pair (Q_4_)	0.65
R_E_ = 0 Ω, C = 12 pF	1	13.5	CM (Q_2_)	0.50
			Diff-pair (Q_4_)	1
R_E_ = 100 Ω, C = 12 pF	1.02	14.8	CM (Q_2_)	0.30
			Diff-pair (Q_4_)	0.50

* Current source structure only. ^x^ Optimized control circuit has a 3-dB bandwidth of about 21 GHz. ^+^ CM: current mirror.
